# Putative founder effect of Arg338* *AP4M1* (SPG50) variant causing severe intellectual disability, epilepsy and spastic paraplegia: Report of three families

**DOI:** 10.1111/cge.14264

**Published:** 2022-12-02

**Authors:** Aurélie Becker, Charlotte Felici, Laëtitia Lambert, Anne de Saint Martin, Marie‐Thérèse Abi‐Warde, Elise Schaefer, Christian Zix, Mina Zamani, Saeid Sadeghian, Jawaher Zeighami, Tahereh Seifi, Reza Azizimalamiri, Gholamreza Shariati, Hamid Galehdari, Mareike Selig, Can Ding, Sarah Duerinckx, Isabelle Pirson, Marc Abramowicz, Guillemette Clément, Bruno Leheup, Philippe Jonveaux, Geneviève Lefort, Myriam Bronner, Mathilde Renaud, Céline Bonnet

**Affiliations:** ^1^ CHRU de Nancy, Laboratoire de Génétique, Inserm U1256, Université de Lorraine Nancy France; ^2^ CHRU de Nancy, Pôle Enfants, Service de Génétique Clinique Nancy France; ^3^ Inserm U1256, Université de Lorraine Nancy France; ^4^ Hôpital Hautepierre, Centre de référence des épilepsies rares Strasbourg France; ^5^ HôpService de Génétique médicale, institut de Génétique médicale d'Alsace, CHU Strasbourg Strasbourg France; ^6^ Hôpital de Forbach Pédiatrie France; ^7^ Department of Biology, Faculty of Science Shahid Chamran University of Ahvaz Ahvaz Iran; ^8^ Narges Medical Genetics and Prenatal Diagnosis Laboratory Ahvaz Iran; ^9^ Department of Pediatric Neurology, Golestan Medical, Educational, and Research Center Ahvaz Jundishapur University of Medical Sciences Ahvaz Iran; ^10^ Department of Medical Genetics, Faculty of Medicine Ahvaz Jundishapur University of Medical Sciences Ahvaz Iran; ^11^ Institute of Human Genetics, University Medical Center of the Johannes Gutenberg University Mainz Germany; ^12^ IRIBHM, Université Libre de Bruxelles Brussels Belgium; ^13^ Genetic Medicine and Development, Faculty of Medicine University of Geneva Geneva Switzerland; ^14^ CHRU de Nancy, Service de neurologie Nancy France

**Keywords:** *AP4M1*, founder effect, spastic paraplegia, SPG50

## Abstract

Bi‐allelic variants affecting one of the four genes encoding the AP4 subunits are responsible for the “AP4 deficiency syndrome.” Core features include hypotonia that progresses to hypertonia and spastic paraplegia, intellectual disability, postnatal microcephaly, epilepsy, and neuroimaging features. Namely, *AP4M1* (SPG50) is involved in autosomal recessive spastic paraplegia 50 (MIM#612936). We report on three patients with core features from three unrelated consanguineous families originating from the Middle East. Exome sequencing identified the same homozygous nonsense variant: NM_004722.4(*AP4M1*):c.1012C>T p.Arg338* (rs146262009). So far, four patients from three other families carrying this homozygous variant have been reported worldwide. We describe their phenotype and compare it to the phenotype of patients with other variants in *AP4M1.* We construct a shared single‐nucleotide polymorphism (SNP) haplotype around *AP4M1* in four families and suggest a probable founder effect of Arg338* *AP4M1* variant with a common ancestor most likely of Turkish origin.

## Graphical abstract

In this study we report three new unrelated patients from Middle‐East consanguineous families carrying the previously described NM_004722.4(AP4M1):c.1012C〉T homozygous nonsense variant. We describe their phenotype, and provide evidence that the subjects carrying this variant originate from a common ancestor.

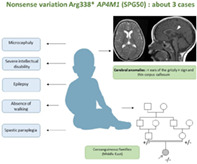

## 1 INTRODUCTION

The clinical phenotypes of patients with variants in any of the four AP4 subunits are very similar and can be grouped together under “AP4 deficiency syndrome.”^1^, ^2^, ^3^ This syndrome of autosomal recessive transmission manifests with intellectual disability and spastic paraplegia.^4^, ^5^

Variants in *AP4M1* gene (MIM *602296) have been identified in several patients.^4^, ^6^, ^7^, ^8^, ^9^ The first homozygous variant was described in 2009.^4^ Recently, in 2020, the description of a large cohort led to defining the clinical, molecular and imaging spectrum of AP‐4‐associated hereditary spastic paraplegia.^5^ In this cohort, *AP4M1*‐associated SPG50 (MIM #612936) was the most common subtype.

In this study we report three new unrelated patients from Middle‐East consanguineous families carrying the previously described NM_004722.4(*AP4M1*):c.1012C>T (rs146262009) homozygous nonsense variant. We describe their phenotype, and provide evidence that the subjects carrying this variant originate from a common ancestor.

## 2 MATERIAL AND METHODS

The patient's parents gave their informed written consent for the publication of clinical data and photographs. The study was carried out in accordance with the Declaration of Helsinki.

Whole‐exome capture was performed with the SureSelect Human All Exon kit (Agilent). The libraries were sequenced using the Ion Proton™ (ThermoFisher), NextSeq 500 (Illumina) and Novaseq 6000 (Illumina) respectively. An in‐house pipeline was used to align FASTQ to the human reference genome (GRCh37/hg19) and to generate VCF. Variants were classified as recommended by the ACMG (American College of Medical Genetics).^10^

Conventional PCR and Sanger sequencing were conducted.

AutoMap v1.0^11^ was used for analysis of homozygous regions from VCF. Haplotypes were created by manual genotyping of a set of SNPs.

## 3 RESULTS

### 3.1 Clinical descriptions

**FIGURE 1:**
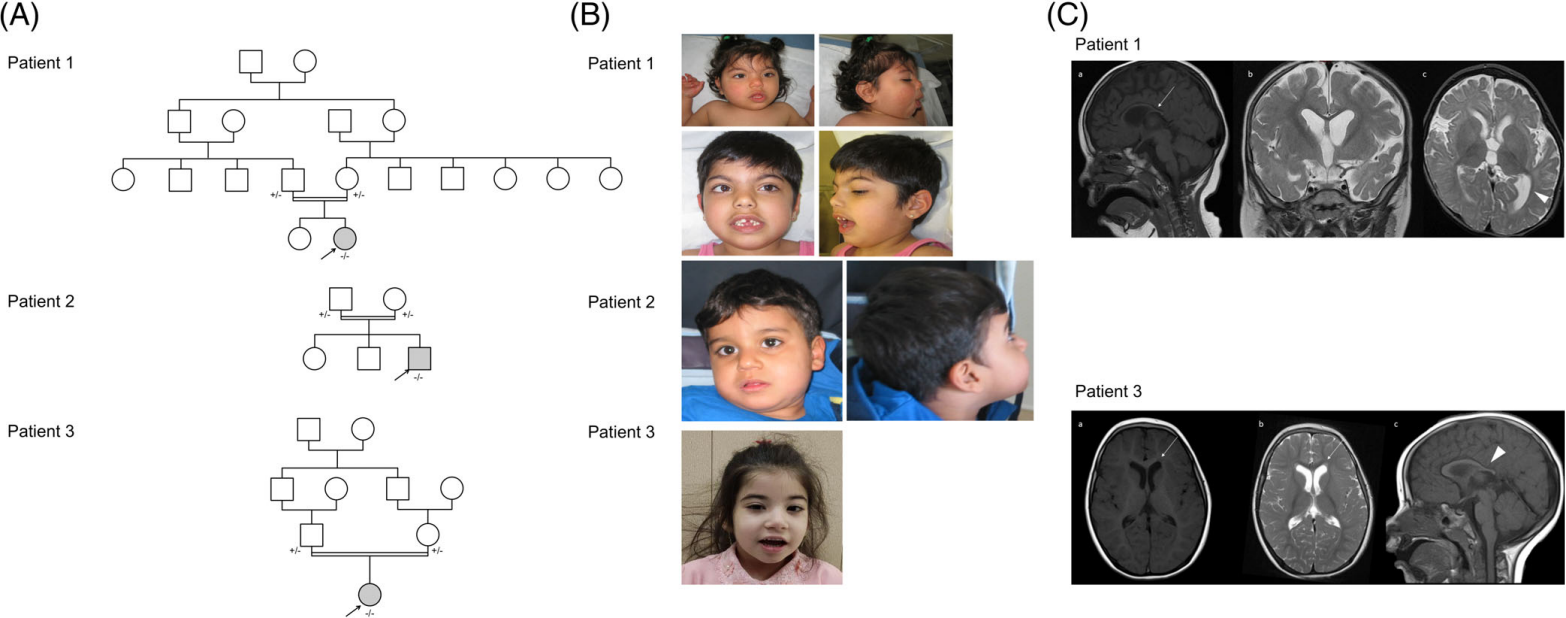
(A) Pedigrees; (B) facial appearance; (C) brain MRIs. Patient 1: sagittal T1‐weighted (A) coronal T2‐weighted (B) sequences: thin splenium of corpus callosum (white arrow), reduction volume in white matter, microcephaly and myelination delay. (C) Axial T2‐weighted sequence: white matter reduction predominating on the left occipital horn (white head arrow), ventriculomegaly with a colpocephalic configuration. Patient 3: (A, B) Axial T1‐weighted (A) and axial T2‐weighted (B) sequences: “ears of the grizzly sign” as a short and round T1 hypointense and T2 hyperintense signal (white arrow). (C) Sagittal T1‐weighted sequence: thin corpus callosum (white head arrow) [Colour figure can be viewed at wileyonlinelibrary.com]

*Patient 1* is an 8‐year‐old girl from a consanguineous Turkish family (Figure 1A and Table 1). Birth head circumference (HC) was 34.5 cm (+0.5SD). She presented early feeding difficulties and global hypotonia. The examination noted early motor delay with head control at 8 months, and sitting unsupported at 13 months. Febrile seizures were described at the age of 11 months. Convulsive seizures were initially generalized and evolved toward a hemicorporeal aspect with transient motor deficit. At the age of 18 months, HC was −2.3SD, and the examination revealed slight dysmorphia including synophris, large mouth and downturned corners of the mouth (Figure 1B). Oculomotor apraxia was present with essentially head rotations. There was right internal strabismus and dystonia of the four limbs. Epilepsy did not respond to valproate. Epilepsy and dystonia were treated by levetiracetam, levodopa and trihexyphenidyl. At the age of 5 years, clinical evaluation reported increased microcephaly (−3.5SD), severe intellectual disability, stereotyped hand movements, no verbal communication, persistent spasticity, and no unsupported walking. Fine‐motor skills were misdirected with choreic movements. Growth was normal. She has been undergoing physical therapy since the age of 7.

**TABLE 1:** Clinical features of patients with bi‐allelic Arg338* *AP4M1* (SPG50) variant

							Development and behavior										Motor System									Seizures			Neuroimaging						
Patient (family)	Sex	Age	Consanguinity	Ethnicity	Microcephaly	Developmental delay	Age at developmental delay	Regression or progressive cognitive	Motor delay	Walking (assisted)	Walking (unsupported)	Non‐ambulatory	Speech delay	Non‐verbal	Shy character	Stereotypic laughter	Neonatal hypotonia	Spasticity	Spasticity ‐ lower extremities	Spasticity ‐ upper extremities	Contractures	Foot deformity	Extrapyramidal Movement disorder	Cerebellar signs	Swallowing Dysfunction/aspiration	Febrile seizures	Epilepsy	Status epilepticus	Thin corpus callosum	Abnormal White Matter signal	Ventriculomegaly	Cerebral atrophy	Cerebellar atrophy	Other neuroimaging findings	Source
1 (1)	F	8y	y	Turkish	y	y	3 m	y	y	y	n	n	y	y	n	n	n	y	y	y	n	n	y	n	n	y	y	‐	y	n	y	y	n	Myelination delay	Present case
2 (2)	M	3y6m	y	Turkish	y	y	0 m	n	y	n	n	y	y	y	n	y	y	y	y	y	y	y	‐	y	y	y	y	y	y	y	y	y	‐	Incomplete myelination, incomplete attachment of the corpus callosum, and dilated internal and external cerebrospinal fluid spaces	Present case
3 (3)	F	5y	y	Arab from Iran	y	y	1 m	n	y	y	n	n	y	n	n	n	n	y	y	y	y	n	n	n	n	y	y	n	y	y	n	n	n	Periventricular white matter T2 hyper signal, ears of grizzly sign	Present case
13 (10)^a^	F	17y	y	Turkish	y	y	‐	y	y	y	n	n	y	n	n	y	‐	y	y	y	n	n	‐	‐	‐	n	y	‐	y	n	y	n	‐		Tuysuz et al^7^; Ebrahimi‐Fakhari et al^5^
14 (10)^a^	F	11y	y	Turkish	y	y	‐	y	y	y	n	n	y	n	n	y	y	y	y	y	n	n	‐	‐	‐	y	y	‐	y	n	y	n	‐		Tuysuz et al^7^; Ebrahimi‐Fakhari et al^5^
42 (25)^a^	M	11y9m	y	Turkish	y	y	4 m	y	y	y	n	y	y	y	n	n	y	y	y	y	y	y	n	‐	y	y	y	y	y	n	y	y	n		Duerinckx^9^; Ebrahimi‐Fakhari et al^5^
110 (70)^a^	F	12y11 m	y	Turkish	y	y	7 m	n	y	y	n	n	y	y	n	n	n	y	y	n	y	y	y	n	‐	n	y	n	y	n	n	n	n		Ebrahimi‐Fakhariet al^5^
Frequency (%) for p.Arg338^a^			100		100	100		67	100	86	**0**	29	100	57	0	29	50	100	100	86	57	43	50	25	50	71	**100**	50	100	29	71	43	0		
Frequency (%) for other variations			77		86	100		51	100		**34**		100		32	50	90	98								70	**62**	24	92	60					Calculated from Ebrahimi‐Fakhari et al^5^

*Note*: y, yes; n, no; ‐, not available. Tüysüz et al.^7^ Ebrahimi‐Fakhari et al.^5^ Duerinckx et al.^9^

^a^ For previous reported cases: patient and family numbering from Ebrahimi‐Fakhari et al.^5^

Brain MRI (17 months) revealed thin splenium of corpus callosum, reduction volume in white matter, microcephaly, ventriculomegaly in a colpocephalic configuration and moderate myelination delay (Figure 1C).

*Patient 2* is a 6‐year‐old boy born to consanguineous Turkish parents (Figure 1A). After birth muscle hypotonia and a HC in the lower range (32 cm, −2SD) were observed. At 1 month microcephaly was already noted (−2.56SD). Between the age of 10 and 12 months the boy developed grand‐mal epilepsy requiring anti‐seizure medications. He remains seizure‐free on medication. At the age of 3 years, he presented with global development delay. He was non‐verbal, but understood simple commands. Episodes of stereotypic laughter were noted. HC was at −4.42SD. He had slight dysmorphic features with a prominent forehead, slightly enlarged earlobes, wide nostrils and downturned corners of the mouth (Figure 1B). He could sit, but neither stood nor walked independently. He had spasticity in all limbs. At the age of 6 years, the boy can move with his wheelchair and spell two words. Independent walking is however not achieved. Early support and physiotherapy are still very useful. Height and weight were normal.

Brain MRI revealed abnormal white matter signal, incomplete myelination, incomplete attachment of the corpus callosum, dilated internal and external cerebrospinal fluid spaces.

*Patient 3* is a 5‐year‐old girl from a consanguineous Arab family (Figure 1A). Birth HC was 35 cm (+1SD). The girl presents with early developmental delay (head control at 18 months, unsupported sitting at 28 months, no independent walking), febrile seizures, reactive epilepsy, contractures in upper limbs, scissor gait, toe walking, flat foot, and back knee. Epilepsy was treated by levetiracetam from infancy until 5 years but after 1 year with no seizure, treatment was withdrawn. She had slight dysmorphic features with epicanthal folds and downturned corners of the mouth (Figure 1B). She is microcephalic (HC: 47 cm [−2.5SD] at 5 years of age). Other growth parameters were normal. The patient has been receiving occupational therapy and speech therapy. Brain MRI showed thin corpus callosum, “ears of the grizzly sign”^5^ as a short and round T1 hypointense signal and T2 hyperintense signal in the forceps minor of the corpus callosum (Figure 1C).

### 3.2 Molecular analysis

NM_004722.4(*AP4M1*):c.1012C>T p.Arg338* homozygous nonsense variant located at chr7:99703901(GRCh37/hg19) was identified in the three patients (ClinVar submission SCV002547281). Sanger sequencing confirmed this homozygous variant and parents are heterozygous. The variant is a stop gain predicted to undergo NMD (Pathogenic Very Strong 1). Allele frequency is extremely low in the GnomAD^12^ database (Pathogenic Moderate 2). ClinVar reports this variant as pathogenic (IDs: 209980) (Pathogenic Supporting 5). The patient's phenotype is quite specific to the *AP4M1* gene (PP4). Our variant is thus classified as “pathogenic” (class 5).

### 3.3 SNP analysis

For patients 1 and 3, we identified a homozygous region of 3.06 Mb containing *AP4M1*. For patient 2, homozygous region size was 12.64 Mb. Haplotype construction (Table 2) showed that four families including a previously reported family^9^ share a common SNP haplotype of 11 SNPs. The shared segment between patients 1 and 3 is larger (98 SNPs) and includes a relatively rare SNP (rs371728730), thus indicating that patients 1 and 3 are genetically closer to the most recent common ancestor.

**TABLE 2:** SNP marker haplotypes of the three patients presenting the *AP4M1* c.1012C>T variation

Chr	Position (GRCh37/hg19)	refSNP	REF	ALT	Patient 1	Patient 2	Patient 3	Duerinckx^9^	gnomAD v3.1.2 frequency of alt allele: All populations	gnomAD v3.1.2 frequency of alt allele: Middle eastern population	Cumulative probability of alleles present in patients haplotype: All populations	Cumulative probability of alleles present in patients haplotype: Middle eastern population
7	99 501 313	rs2572003	A	T	AF = 0.00	AF = 0.00	AF = 1.00	nd	‐	‐	‐	‐
7	99 654 600	rs11558475^a^	A	G	AF = 1.00	AF = 0.00	AF = 1.00	AF = 1.00	‐	‐	‐	‐
7	99 654 689	rs11558476^a^	G	A	AF = 1.00	AF = 0.00	AF = 1.00	AF = 1.00	‐	‐	‐	‐
7	99 686 873	rs4729575^a^	G	T	AF = 1.00	AF = 0.00	AF = 1.00	nd	‐	‐	‐	‐
7	99 691 740	rs1527423^a^	G	A	AF = 1.00	AF = 0.00	AF = 1.00	AF = 1.00	‐	‐	‐	‐
7	99 692 993	rs2261360^a^	G	T	AF = 1.00	AF = 0.00	AF = 1.00	AF = 1.00	‐	‐	‐	‐
7	99 693 078	rs12267^a^	G	A	AF = 1.00	AF = 0.00	AF = 1.00	AF = 1.00	‐	‐	‐	‐
7	99 696 370	rs1534309^a^	C	G	AF = 1.00	AF = 0.00	AF = 1.00	nd	‐	‐	‐	‐
7	99 699 436	rs1122598	G	A	**AF = 1.00**	**AF = 1.00**	**AF = 1.00**	**AF = 1.00**	0,1572	0,2962	15,72%	29,62%
7	99 699 626	rs2293481	T	C	**AF = 1.00**	**AF = 1.00**	**AF = 1.00**	**AF = 1.00**	0,5843	0,6582	9,19%	19,50%
7	99 701 176	rs999885	G	A	**AF = 1.00**	**AF = 1.00**	**AF = 1.00**	**AF = 1.00**	0,4744	0,5759	4,36%	11,23%
7	99 701 640	rs4729577	T	C	**AF = 1.00**	**AF = 1.00**	**AF = 1.00**	**AF = 1.00**	0,5846	0,6614	2,55%	7,43%
7	99 703 901	rs146262009^b^	C	T	**AF = 1.00**	**AF = 1.00**	**AF = 1.00**	**AF = 1.00**	0,00003943	0,0000	‐	‐
7	99 703 958	rs2293479	T	C	**AF = 0.00**	**AF = 0.00**	**AF = 0.00**	**AF = 0.00**	0.2687	0.1592	1,86%	6,24%
7	99 704 796	rs13309	A	T	**AF = 1.00**	**AF = 1.00**	**AF = 1.00**	**AF = 1.00**	0,5639	0,6487	1,05%	4,05%
7	99 704 827	rs1050542	A	G	**AF = 1.00**	**AF = 1.00**	**AF = 1.00**	**AF = 1.00**	0,5397	0,6424	0,57%	2,60%
7	99 707 712	rs2272338	A	G	**AF = 1.00**	**AF = 1.00**	**AF = 1.00**	**AF = 1.00**	0,5846	0,6551	0,33%	1,70%
7	99 707 950	rs4134917	C	T	**AF = 1.00**	**AF = 1.00**	**AF = 1.00**	nd	0,5841	0,6551	0,19%	1,12%
7	99 710 584	rs4134904	A	AG	**AF = 1.00**	**AF = 1.00**	**AF = 1.00**	**AF = 1.00**	0,5842	0,6592	0,11%	0,74%
7	99 725 216	rs371728730^a^ ^,^ ^c^	C	A	AF = 1.00	AF = 0.00	AF = 1.00	AF = 1.00	*0,009302*	*0,02229*	0,00%	0,02%
7	99 747 130	rs12878^a^	G	A	AF = 1.00	AF = 0.00	AF = 1.00	AF = 1.00	‐	‐	‐	‐
7	99 751 017	rs3736591^a^	A	G	AF = 1.00	AF = 0.00	AF = 1.00	AF = 1.00	‐	‐	‐	‐
7	99 751 281	rs3736590^a^	G	T	AF = 1.00	AF = 0.00	AF = 1.00	AF = 1.00	‐	‐	‐	‐
7	99 752 566	rs55839153^a^	G	A	AF = 1.00	AF = 0.00	AF = 1.00	nd	‐	‐	‐	‐
7	99 753 121	rs2272337^a^	A	G	AF = 1.00	AF = 0.00	AF = 1.00	nd	‐	‐	‐	‐
7	99 757 612	rs3823646^a^	G	A	AF = 1.00	AF = 0.00	AF = 1.00	AF = 1.00	‐	‐	‐	‐
7	*72 consecutive SNPs from rs3800951 to rs3087504*				AF = 1.00	‐	AF = 1.00	nd				‐
7	100 486 656	rs13241786	T	G	AF = 1.00	‐	AF = 1.00	AF = 1.00	‐	‐	‐	‐
7	100 547 281	rs73163738	C	A	AF = 0.50	‐	AF = 0.00	nd	‐	‐	‐	‐

*Note*: REF: reference allele; ALT: alternative allele; AF: allelic fraction of alternative allele, AF = 1.00 if alternative allele is present on both chromosomes, AF = 0.50 if alternative allele is present on one of the chromosomes, AF = 0.00 if reference allele is present on both chromosomes; bold: common haplotype; nd: no data.

^a^ Different haplotype in at least one of the three patients.

^b^ NM_004722.4(AP4M1):c.1012C>T p.Arg338*.

^c^ Frequency <1%.

## 4 DISCUSSION

Exome sequencing in three patients with severe intellectual disability, microcephaly, epilepsy, dystonia, spasticity and cerebral atrophy revealed the same homozygous substitution p.Arg338* in *AP4M1*.

This variant was described for the first time in two affected sisters of Turkish origin born of a consanguineous union.^7^ This variant was subsequently reported in a patient from a consanguineous Turkish family.^9^ A sixth consanguineous Turkish family was recently described.^5^

A total of 18 different pathogenic variants in *AP4M1* are listed in LOVD database and 23 in the largest published cohort.^5^ The majority of variants are nonsense or frameshift. They cover most exons of the gene. It is reported that despite some clinical variability, there is similarity in the core clinical features in AP4 deficiency syndromes indicating that the underlying mechanism is probably partial loss of the AP4 subunit.^5^

We compared phenotypes associated with Arg338* and other *AP4M1* variants.^5^ Patients share a similar phenotype concordant with “AP4 deficiency syndrome.” However, although differences are not significant due to insufficient patient numbers, epilepsy was present in 100% of patients (vs. 62% for other variants), unsupported walking was never achieved for 100% of patients (vs. 66%) and patients are non‐verbal (Table 1). Motor symptoms are progressive and true regression is often described.^5^ However, despite their relative young age (8, 6 and 5 years old), our three patients already present spasticity. Cerebral atrophy which is mainly described in advanced disease^5^ are already observed in patients 1 and 2 (8 and 6 years old). Regarding treatments, antiepileptic drugs and physical therapy are very useful.

We emphasize that neuroimaging is a key element in diagnosis. As recently demonstrated, corpus callosum and periventricular white matter abnormalities are highly sensitive findings in AP4 deficiency^13^ and provide guidance for etiological diagnosis prior to sequencing. Knowledge of these signs should help to guide diagnosis and reduce diagnostic time.

*AP4M1* variants are described in a wide range of different ethnicities and consanguinity is reported in two‐thirds of patients, however the majority of variants are private.^5^ Recurrence of Arg338* *AP4M1* variant in at least six consanguineous families from the Middle East is suggestive of a founder effect. We have shown that four families shared a common SNP haplotype, highlighting a likely founder effect.

Patients 1 and 2 are Turkish while patient 3 originates from the Iranian Arab population which constitutes around 3% of Iran's population and has intermingled with Turks.^14^ Iranome database^15^ showed an allele frequency of 2/1600 for Arg338* *AP4M1* variant. GnomAD^12^ allele frequency is 8/128902 in European population. Thus, allele frequencies are in favor of a founding effect in Turkish population. Accordingly, patients with relevant clinical features of Turkish origin should be considered as potential candidate for this variant.

In summary, we describe the first case series of SPG50 patients carrying Arg338* *AP4M1* variant. We suggest that the families with Arg338* *AP4M1* variant probably have a common Turkish ancestor.

## AUTHOR CONTRIBUTIONS

Material preparation, data collection and analysis were performed by Aurélie Becker, Charlotte Felici and Céline Bonnet. Charlotte Felici wrote the first draft if the manuscript. Céline Bonnet supervised the project and edited the manuscript. All authors approved the final manuscript.

## CONFLICT OF INTEREST

The authors declare no conflict of interest.

### PEER REVIEW

The peer review history for this article is available at https://publons.com/publon/10.1111/cge.14264.

## Data Availability

The data that support the findings of this study are available from the corresponding author upon reasonable request.
